# Identification and Prioritization of the Economic Impacts of Vaccines

**DOI:** 10.1155/2016/6267343

**Published:** 2016-12-12

**Authors:** Ingeborg M. van der Putten, Aggie T. G. Paulus, Silvia M. A. A. Evers, Raymond C. W. Hutubessy, Mickael Hiligsmann

**Affiliations:** ^1^CAPHRI, School of Public Health and Primary Care, Department of Health Services Research, Maastricht University, Maastricht, Netherlands; ^2^Initiative for Vaccine Research, World Health Organization, Geneva, Switzerland

## Abstract

Understanding the most important economic impacts of vaccines can provide relevant information to stakeholders when selecting vaccine immunization strategies from a broader perspective. This study was therefore designed to first identify economic impacts to vaccinated individuals and, second, assess the relative importance of these economic impacts. A four-step approach was used, including a review of the literature, a pilot study, and expert consultation. As a fourth step, a survey utilizing a best-worst scaling was conducted among 26 different stakeholders to assess the relative importance of the identified economic impacts. In each of the 15 choice tasks, participants were asked to choose the most important and the least important economic impact from a set of four from the master list. We identified 23 economic impacts relevant for vaccine introduction. Four domains were identified, namely, health related benefits to vaccinated individuals, short- and long-term productivity gains, community or health systems externalities, and broader economic indicators. The first domain was seen as especially important with mortality, health care expenditure, and morbidity ranking in the top three overall. In conclusion, our study suggests that domain A “health related benefits to vaccinated individuals” are valued as more important than the other economic impacts.

## 1. Introduction

Globally, the introduction of vaccines has had an important impact on the reduction of communicable diseases [1]. This can be seen in the reduction of the under-5 mortality rate from 12.7 million children in 1990 to 6.3 million children in 2013 [2]. Currently, it is estimated that immunization averts 2 to 3 million deaths every year [3]. Furthermore, vaccination is believed to be one of the most cost-effective interventions [4]. Consequently, many countries have introduced national vaccine programs, often referred to as an Expanded Program of Immunization (EPI), to prevent the most common and deadly diseases. However, such programs are not static but are developing over time.

Before a new or improved vaccine is added to such a program, the vaccine first has to be evaluated. In this process called vaccine introduction, one of the steps is to look at the economic and financial consequences of introducing the vaccine [5]. This step has become increasingly important as government resources have become less available. As a result, governments have to choose among many competing interventions inside and outside the healthcare sector [6]. For instance, governments must decide whether they want to invest in free primary education, sanitation programs, housing, vaccination, or other healthcare interventions.

For vaccine introduction decisions, the following economic and financial considerations are important. First, stakeholders are in need of decision-supportive information, such as the outcomes of economic evaluations [7–9]. Second, to expand the budget available for vaccines, instead of merely redistributing the existing budget, other stakeholders get involved, such as people from the department of finance, parliamentarians, civil society, and media [10].

Currently, many economic evaluations of immunization strategies concentrate on immediate health gains and household cost-savings. However, to better inform all the stakeholders involved in vaccine introduction decisions, outcomes should also include the broader economic impacts of vaccines, which have long-term effects [11, 12]. These effects are experienced by those who are vaccinated and society as a whole, including nonvaccinated community members [13].

Several attempts to provide frameworks for the broader economic impacts of vaccines have been performed [13–15]. However, it is largely unknown how stakeholders value these kinds of economic impacts [16] (i.e., which impacts do stakeholders find most and least important). van der Putten et al. (2015) tested an initial framework to gain insight into the viewpoints of different stakeholders in low and middle income countries (LMIC) [17]. In this mixed-method study, it was found that the broader economic impact of vaccines was seen to be as important as measuring the burden of disease. However, this framework needed to be updated to incorporate new insights that were developed in the field [13–15, 18] and further validation among a larger group of different stakeholders. In addition, it was suggested that different audiences need different messages because of differences in culture and context [17]. Furthermore, a more detailed prioritization of the economic impacts of vaccines would be important to steer research investments into the most valued areas.

To assess the broader economic impact of vaccination and prioritize different types of economic impacts, rankings are needed by different stakeholders, such as policy makers, providers of immunization services, health advocates, and researchers. Given the abovementioned limitations of prior studies and the importance of gaining insight into the preferences of different stakeholders, this study was designed with the aim to (1) identify what kind of economic information could be important for stakeholders when making decisions on introducing vaccines in general; (2) assess the relative importance of economic impacts of vaccines by ranking them as most important and least important in a best-worst scaling; (3) find out if context (availability and relevance), type of stakeholder group, or working in a low and middle income countries (LMIC) versus high income countries (HIC) influence preferences stated.

## 2. Methods

**Figure 1:**
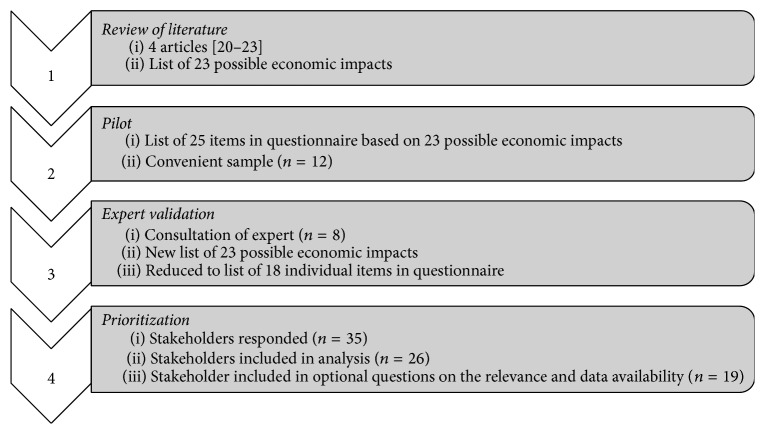
Steps in the identification and prioritization of the economic impacts.

To identify and prioritize the broader economic impact of vaccines, a four-step process was used (as shown in Figure 1). The first three steps were undertaken to come up with a renewed framework for the economic impacts of vaccines, while the fourth step prioritized the identified economic impacts using a best-worst scaling.

### 2.1. Identification

In the first step, we reviewed the literature and used the outcomes of several papers, including three literature reviews [13–15] and a mixed-method study [17], to identify a list of the economic impacts of vaccines. The mixed-method study consisted of a survey among participants of the New and Underutilized Vaccine Initiative (NUVI) meeting 2011 (*n* = 26) and interviews with stakeholders (*n* = 14) [17]. More detailed descriptions of the methods can be found in the referenced articles [13–15, 17]. A total of 23 different short-term effects of vaccines, long-term effects of vaccines, and the effects experienced by society as a whole were included in the framework. This framework was first used to design a version of the questionnaire, which consisted of 25 individual items. Then, this version was pilot tested by a convenient sample of 12 colleagues. The pilot revealed that the questionnaire was too time consuming. Furthermore, the comparison of the different types of impacts was difficult for several reasons: the questionnaire consisted of too many items; the used economic impacts were not all from the same level; and the used impacts were not totally exclusive. To overcome these problems, we decided to add a third step to validate the framework using individual consultations with experts (*n* = 8) via Skype or telephone interviews. In these interviews, we provided the experts with the initial framework and asked them if all included economic impacts were relevant, if some economic impacts could be combined, if any economic impacts should be added, and if the proposed items for the survey were correctly formulated. Based on these results, a renewed framework of 23 economic impacts of vaccines was formulated. In a consensus meeting with the authors, three criteria were established to formulate the final questionnaire: (1) all items should be measured on the same level; (2) items should not overlap; and (3) it should be possible to calculate the item in monetary terms. As a result, the final framework consisted of 18 items for the questionnaire. An overview of how the framework of 23 economic impacts of vaccines was reduced to 18 individual items in Supplementary Material available online at http://dx.doi.org/10.1155/2016/6267343.

### 2.2. Prioritization

To prioritize the 18 individual items, a survey was conducted (fourth step). Policy makers, providers of immunization services, health advocates, researchers, and other participants were actively recruited by using personal contacts, the Supporting Independent Immunization and Vaccine Advisory Committees (SIVAC) Initiative database, internet searches for collaborative initiatives for vaccines, among participants at the WHO Broad Economic Impact of Vaccines Meeting (24-25 November 2014, Bangkok, Thailand) and Annual European Congress, ISPOR, (8–12 November 2014, Amsterdam, Netherlands), World Health Forum (22–24 November 2015, The Hague), Advancing The Value Of Vaccines Research Agenda (26-27 April 2016, Boston, US,), and snowballing via personal contacts, the contacts of the eight experts, and the participants at the meeting in Bangkok and the meeting in Boston.

The questionnaire consisted of four parts: some background questions, a rating scale exercise (RSE), a best-worst scaling exercise (BWS), and an optional question on the relevance and data availability of the identified economic impacts. For each identified economic impact, a description was developed that was pilot tested in step two. The descriptions of all the economic impacts of vaccines were included in the introduction part of the questionnaire. With the RSE, the priority of the different identified economic impacts was then established using a 0–10 VAS scales to familiarize respondents with the different economic impacts. A BWS was used to further assess the relative importance of these economic impacts. A BWS simplifies the ranking task for respondents by reducing the number of choices they have to make by asking them to indicate the best and worst option among a list of factors. Advantages of this method are robustness for scale-related biases and effective discrimination between ratings of different factors involved in complex decisions [19]. Therefore, we chose to present only the outcomes of the BWS in the Results.

Respondents were asked to answer 15 choice sets, each composed of a set of 4 factors from the master list of 18 individual items (see Table 1). In each choice set, respondents were asked to identify the most important and the least important economic impact (an example of a BWS task is given in Table 2). We used Sawtooth Software's SSI Web platform (version 8.3) to design the BWS questions. In order to create an efficient design, the following measures were taken. First, each item in the total six versions of the questionnaire was shown 20 times, displayed 4 to 6 times in every position, and paired 3 to 4 times with every other item on the master list. No sets were shown twice and questions were divided into 6 different versions of the questionnaire to reduce context bias [20]. The six versions of the questionnaire were randomly assigned to the respondents via online questionnaire software [21].

Only respondents who filled out the BWS questions were included in the analysis. Descriptive analysis was performed on the background questions regarding work experience, type of stakeholder, and self-reported knowledge on economic evaluations, policy decisions, and vaccines. A Hierarchical Bayes (HB) analysis was performed to analyze the BWS questions using Sawtooth Software. This software uses HB modeling [22] to calculate raw scores and relative importance score (RIS) of each item on the master list. All 26 respondents were included in the calculations as all scored above the 0.282 fit statistic [23]. In this research, only the RIS scores are used. These scores indicate the likelihood of one item being selected and sums up to 100% [23].

Subgroups analyses were conducted for stakeholders working in LMIC and HIC and between stakeholders with a research background and other stakeholders. One-way ANOVA tests were conducted to assess statistical differences between groups [24]. If normality assumption was not met, Mann–Whitney* U* nonparametric tests were executed [24].

## 3. Results

### 3.1. Identification

In total, 23 economic impacts of vaccines were identified after the expert consultation round (steps (1)–(3)). With the input of the consultations with the experts, a final list of 18 items to be included in the survey was made during the consensus meeting (see Table 1). The 18 items can be subdivided into four different domains [15]. The first domain (A) (3 items), “health related benefits to vaccinated individuals,” consists of health gains and healthcare cost-savings. The second domain (B) (3 items) includes “short-term and long-term productivity gains.” These gains refer to the individual (long-term) productivity due to better physical and mental health. The third domain (C) (6 items) consists of “community or health systems externalities,” which are related to the decline of prevalence and incidence of vaccine-related diseases and different types of equity considerations. Finally, the fourth domain (D) (6 items) includes “broader economic indicators,” such as the impact of vaccine immunization strategies on GDP and tax revenues.

### 3.2. Prioritization

In total, 35 respondents started the questionnaire, of which 26 respondents were included in the analysis. As the last part of the questionnaire was optional, nineteen respondents answered the questions on the relevance and data availability of the identified economic impacts. The average working experience was thirteen years and most respondents (61.5%) had a research background. Furthermore, two health advocates, five health policy makers, and three providers of immunization services filled out the questionnaire. Fifteen respondents worked in LMIC and ten in HIC. The self-reported knowledge of the respondents was 6.2 (1.4) on economic evaluations, 3.4 (2.0) on decision-making, and 5.9 (1.3) on vaccines measured on a 7-point Likert scale (strongly disagree – strongly agree). The general ranking of the domains was consistent for both RSE and BWS. Also, the ranking of the individual items was quite similar to the ranking of the domains. Results of the RSE, which are available upon request from the first author, were similar to results from the BWS.

#### 3.2.1. Best-Worst Scaling

The BWS was rated 6.2 (SD = 2.4) on a scale of 10 (extremely easy – extremely difficult). Information on the domain A “health related benefits to vaccinated individuals” was ranked first, domain B “short-term and long-term productivity-related gains” ranked second, domain C “community or health system externalities” ranked third, and domain D “broader economic indicators” ranked fourth (see Table 3). Looking at individual items, mortality, healthcare expenditure, and morbidity are statistically the three most important economic impacts since their confidence intervals are not overlapping with other impacts. Almost all the items of domain D “broader economic indicators” scored the lowest except for behavior related productivity, which is ranked fourth. Risk reduction and outbreak prevention costs are seen as more important than the other items of domain C “community or health system externalities,” while school absenteeism is seen as not as important when compared with domain B “outcome related productivity” and “care related productivity.”

#### 3.2.2. Relevance and Data Availability

**Figure 2:**
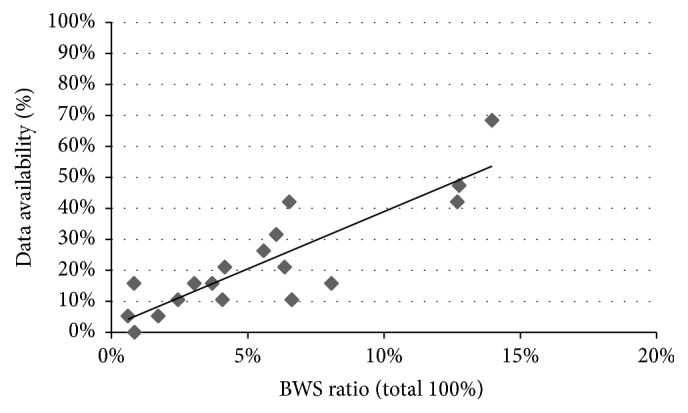
Scatterplot impact of data availability on outcome BWS ratio.

Most respondents found the included individual items relevant and applicable in their countries except for domain D “broader economic indicators” (Table 3). However, Table 3 also shows data on domain A “health related benefits to vaccinated individuals” is available in some countries. This is not the case for data on domain B “short-term and long-term productivity-related gains,” domain C “community or health system externalities,” and domain D “broader economic indicators” where data on these individual items are often unavailable or not used. In Figure 2, a scatterplot of the percentage of respondents that have data available in their country for every economic impact is plotted against the outcomes of the RIS. You can see that known data availability in the country of work of the respondent is positively related to the scores on the BWS ratio.

#### 3.2.3. Subgroups Analyses

**Figure 3:**
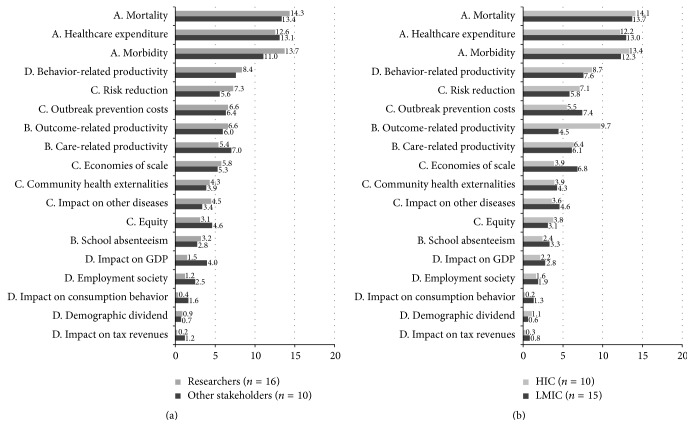
(a) Results of BWS measured on a sum scale of 100% researchers versus other stakeholders. (b) Results of BWS measured on a sum scale of 100% LMIC versus HIC (A: Health related benefits to vaccinated individuals; B: Short-term and long-term productivity gains; C: Community or health systems externalities; D: Broader economic indicators).

For some of the individual items, researchers (*n* = 16) have a different viewpoint than policy makers, providers of immunization services, and health advocates (*n* = 10). Impact on GDP, care related productivity, and equity are seen as more valuable by stakeholders of the latter group while researchers value information on morbidity. None of the individual items were significantly different (see Figure 3(a)).

When looking at the outcomes of respondents working in LMIC (*n* = 15) and respondents working in HIC (*n* = 10), no significant differences could be established for the individual items either, although outcome related productivity was regarded as more important in HIC while economies of scale and outbreak prevention costs are valued more in LMIC (see Figure 3(b)).

## 4. Discussion

The aim of this study was to find out which broad economic impacts of vaccines could be included in economic evaluations to better meet the needs of the previously identified stakeholders, because developing new outcomes for economic evaluations could help stimulate evidence-based decision-making in LMIC and HIC.

This study identified 23 economic impacts that could be relevant for vaccine introduction. The BWS suggested that domain A “health related benefits to vaccinated individuals” is valued as more important than the other domains. Most individual items were seen as relevant, except of several items of domain D “broader economic indicators.” The outcomes of the analysis of the data availability in specific countries suggest a positive relationship with the RIS score. Furthermore, group comparison of the BWS outcomes for different stakeholder groups showed no significant differences.

Many steps were taken to identify the economic impact of vaccines and to develop the questionnaire used. Each step added rigorousness to the design of this study. Selected individual items were checked based on face validity by the 8 experts. The general ranking of the domains was consistent for both RSE and BWS; also the ranking of the individual items was quite similar, which suggests that the outcomes are reliable. Although we used numerous strategies to find the right respondents, one main shortcoming of this study in general is the low response rate. More specifically, health decision-makers inside and outside the healthcare sector were underrepresented. Therefore, results of the subgroup analysis should be interpreted with caution. Moreover, as you need a reasonable number of respondents for calculating the RIS scores to obtain stable results from the analysis, samples smaller than twenty are not recommended [22]. The difficulties in the data collections lead to a relatively long data collection period of 1.5 years. The overrepresentation of researchers in the sample also makes it more difficult to generalize about the preferences attributed to other stakeholder groups involved in the decision-making around vaccine introduction.

Three main findings of this research should be discussed. First, with some minor difference, the framework we propose largely overlaps with alternative frameworks recently published in the literature [15, 18]. One difference is that Bärnighausen et al. choosed to divide the impacts in narrow and broad categories and we opted to use the domain classification of Jit et al. Another difference is that, in the publication of the framework by Bärnighausen et al., health gains were transferred from narrow to broad while we opted to keep mortality and morbidity outcomes in the first domain [18]. Second, the findings of this study are not fully consistent with a study undertaken earlier with a preliminary framework in which we found that the broader economic impacts were as important as the narrow economic impacts [17]. However, this study was also undertaken in a small sample (*n* = 26), and the method used in this study (BWS) has several advantages over Likert scales, as it can be used to better discriminate between the items to be ranked and is less prone to scale-related biases [19]. Third, our study also suggests that information and data on broader impact of vaccines are also less available in countries. This can have several meanings, for example, that information on BEIV is not available as it is not seen as very helpful for policy making or that respondents are not aware of the possibilities of these values and therefore put a lower value on these types of impacts.

On the basis of these results, we recommend looking at broader outcome measures, while retaining the more traditional measures on mortality, morbidity, and healthcare expenditure as the focus of economic evaluations. Furthermore, this study could indicate that it is important to better inform decision-makers with the broader economic impacts of vaccines before making decisions about the usefulness of such economic impacts. Therefore, we recommend developing programs to educate stakeholders on the possibilities of BEIVs and research how to impact the stated preferences of the different groups of stakeholders. To gather these data, we would recommend organizing workshops around this subject during trainings and meetings where all stakeholder groups are already represented. This could be in the form of very short questionnaires or data collection in a workshop setting. Although the framework was initially developed for LMIC [13–15, 17], parts of the framework are also applicable to HIC, to stimulate the introduction of newly developed vaccines especially in HIC with highly variable incomes. For example, introduction decisions on both pandemic and seasonal influenza vaccines and recently registered dengue vaccines may include broader social and economic considerations [25–28].

## 5. Conclusion

This study suggests that domain A “health related benefits to vaccinated individuals” which are traditionally used in economic evaluations is valued as most important by both policy makers and researchers, and in both LMIC and HIC.

## Supplementary Material

Overview (broader) economic impacts of vaccines.

## Figures and Tables

**Table 1:** Lists of all economic impacts of vaccines.

Impacts	Individual item
*(A) Health related benefits to vaccinated individuals*	
(1) Mortality	Health benefits achieved by reducing number of deaths.
(2) Morbidity	Health benefits achieved by reducing morbidity and improving quality of life.
(3) Healthcare expenditure	Reduction in medical expenditures for healthcare system.

*(B) Short-term and long-term productivity gains*	
(4) School absenteeism	Reduction in amount of schooldays missed due to illness.
(5) Care-related productivity	Increased individual productivity due to reduction in lost working days.
(6) Outcome-related productivity	Increased individual lifetime productivity and participation due to improved health.

*(C) Community or health systems externalities*	
(7) Impact on other diseases	Impact on incidence numbers of closely related diseases not vaccinated for.
(8) Community health externalities	Externalities among the unvaccinated community members.
(9) Outbreak prevention costs	Impact on disease outbreak investigations and prevention.
(10) Equity	Impact on equity issues in the society.
(11) Risk reduction	Impact on welfare of households due to reduced uncertainty in future outcomes and health expenditures.
(12) Economies of scale	Impact on per dose price of vaccine due to changes in demand.

*(D) Broader economic indicators*	
(13) Behaviour-related productivity	Economic benefits for families as a result of improved child health and survival.
(14) Demographic dividend	Economic effects of changes in demographic composition of society.
(15) Employment in society	Impact on overall employment in society.
(16) Impact on consumption behaviour	Impact on the consumption of the general population.
(17) Impact on gross domestic product (GDP)	Impact on gross domestic product in general.
(18) Impact on tax revenue	Impact on tax revenues.

**Table 2:** Example of a best-worst scaling question.

Most important		Least important
	Equity considerations	X
	Economic information	
	Effectiveness vaccines	
X	Mortality rates	

This expert indicated that in this scenario “mortality rates” are the most important impact and “equity considerations” the least important impact in their decision-making process.

**Table 3:** Overview ranking BWS, relevance, and data availability.

Economic impact (domain)	RIS (95% CI) *n* = 26	Relevance (%) *n* = 19	Data availability (%) *n* = 19
(1) Mortality (A)	13.96 (12.88–15.04)	17 (89.5%)	13 (68.4%)
(3) Healthcare expenditure (A)	12.76 (11.43–14.09)	15 (78.9%)	9 (47.4%)
(2) Morbidity (A)	12.69 (11.53–13.85)	19 (100%)	8 (42.1%)
(13) Behavior-related productivity (D)	8.07 (6.73–9.41)	14 (73.7%)	3 (15.8%)
(11) Risk reduction (C)	6.62 (4.64–8.59)	13 (68.4%)	2 (10.5%)
(9) Outbreak prevention costs (C)	6.52 (4.46–8.59)	16 (84.2%)	8 (42.1%)
(6) Outcome-related productivity (B)	6.35 (4.60–8.10)	16 (84.2%)	4 (21.1%)
(5) Care-related productivity (B)	6.05 (4.51–7.59)	17 (89.5%)	6 (31.6%)
(12) Economies of scale (C)	5.59 (3.57–7.60)	15 (78.9%)	5 (26.3%)
(8) Community health externalities (C)	4.15 (2.93–5.37)	17 (89.5%)	4 (21.1%)
(7) Impact on other diseases (C)	4.07 (2.54–5.61)	14 (73.7%)	2 (10.5%)
(10) Equity (C)	3.69 (1.93–5.45)	11 (57.9%)	3 (15.8%)
(4) School absenteeism (B)	3.04 (2.25–3.83)	17 (89.5%)	3 (15.8%)
(17) Impact on GDP (D)	2.44 (1.13–3.74)	8 (42.1%)	2 (10.5%)
(15) Employment in society (D)	1.72 (0.73–2.70)	8 (42.1%)	1 (5.2%)
(16) Impact on consumption behavior (D)	0.85 (0.00–3.75)	7 (36.8%)	0 (0.0%)
(14) Demographic dividend (D)	0.82 (0.34–1.31)	8 (41.2%)	3 (17.6%)
(18) Impact on tax revenues (D)	0.60 (0.13–1.07)	7 (35.3%)	1 (5.9%)

A: Health related benefits to vaccinated individuals. B: Short-term and long-term productivity gains. C: Community or health systems externalities. D: Broader economic indicators.
