# Supplementary material for: Identification and Prioritization of the Economic Impacts of Vaccines
Source: Biomed Res Int. 2016 Dec 12;2016:6267343. doi: 10.1155/2016/6267343 (PMC5183751; doi:10.1155/2016/6267343)
Supplement: Supplementary file 1 — Overview (broader) economic impacts of vaccines. [file 6267343.f1.pdf]

Overview (broader) economic impacts of vaccines

Definition: Broader economic impacts can described as longer term effects that go beyond individuals vaccinated and their caregivers. They can be expressed in both costs and benefits for another party, such as the society or community.

Value	Found explanation in literature	Value Survey	Item in survey
A. Health related benefits to vaccinated individuals			
**1a. Health gains - mortality**	Reduction in mortality or morbidity through vaccination presented in natural units of health. Natural units of health include number of deaths or disability, years of life saved, cases of illness, quality adjusted life years (QALY) or disability adjusted life years (DALY), which are not presented in dollar units [1]	**1. Mortality**	Health benefits achieved by reducing number of deaths.
**1b. Health gains - morbidity**		**2. Morbidity**	Health benefits achieved by reducing morbidity and improving quality of life.
**2. Health care cost savings**	Savings of medical expenditures, health care system savings, and household savings because vaccination prevents illness episodes [1, 2]	**3. Health care expenditure**	Reduction in medical expenditures for health care system.
B. Short-term and long-term productivity gains			
**3. School absenteeism**	Amount of schooldays missed due to illness [3, 4]	**4. School absenteeism**	Reduction in amount of schooldays missed due to illness.
**4. Care-related productivity**	Savings of parents' productive time because vaccination avoids the need for taking care of a sick child [1, 2]	**5. Care-related productivity**	Increased individual productivity due to reduction in lost working days.
**5. Outcome-related productivity gains**	Increased productivity from averted mortality and morbidity, including the productivity benefits from improved cognition and physical strength, as well as school enrolment, attendance and attainment [1, 2]	**6. Outcome-related productivity**	Increased individual lifetime productivity and participation due to improved health.
C. Community or health system externalities			
**6. Serotype replacement effects & cross protection**	Impact on incidence numbers of closely related diseases not vaccinated for [5].	**7. Impact on other diseases**	Impact on incidence numbers of closely related diseases not vaccinated for.
**7. Community health externalities**	Externalities among the unvaccinated community members.<br>- Herd effects are reductions in unvaccinated persons' risk of contracting disease due to the vaccination of others. Herd effects occur because vaccinated individuals will not contract and transmit a disease	**8. Community health externalities**	Externalities among the unvaccinated community members.

Value	Found explanation in literature	Value Survey	Item in survey
	between infected and susceptible individuals, reducing disease transmission in a population [5].<br>- Vaccination can prevent disease and thus obviate the need for antibiotic use, reducing the prevalence of antibiotic-resistant strains [5].		
**8. Outbreak-prevention costs**	Impact on disease outbreak investigations and prevention [1]	**9. Outbreak prevention costs**	Impact on disease outbreak investigations and prevention.
**9. Equity**	The absence of avoidable or remediable differences among groups of people, whether those groups are defined socially, economically, demographically, or geographically. [6]	**10. Equity**	Impact on equity issues in the society.
**10. Risk reduction**	Protection to households from uncertainty in future outcomes, such as catastrophic health expenditure due to chronic illness and/or long-term disability [1]	**11. Risk reduction**	Impact on welfare of households due to reduced uncertainty in future outcomes and health expenditures.
**11. Capacity building**	Education and training of healthcare workers improved managerial skills of healthcare workers [7]		
**12. Platform for other interventions**	Entry point for providing a wide range of other primary health care services such as family health education [7]		
**13. Health resources**	Impact of vaccine programs on amount of health resources available e.g., Extra vehicles and new 'ambassadors' for advocacy activities [7]		
**14. Priority of interventions**	Overlooking importance of social determinants of health by focusing on 'silver bullets' and 'mass campaigns' instead of adapting interventions to the prevailing culture and socioeconomic conditions, which generate the felt needs. [8]		
**15. Creating demand for vaccines and potential for partial cost recovery**	Vaccinees may be willing to pay a small amount towards the cost of vaccination if the government is able to subsidize most of the cost, hence enabling partial cost recovery [9]		
**16. Economies of scale**	Impact on per dose price of vaccine due to changes in demand [9].	**12. Economies of scale**	Impact on per dose price of vaccine due to changes in demand.
D. Broader economic indicators			
**17. Behaviour-related productivity gains**	Benefits accruing because vaccination improves child health and survival and thereby changes household	**13. Behaviour-related productivity**	Economic benefits for families as a result of improved child health and survival.

Value	Found explanation in literature	Value Survey	Item in survey
	choices, such as fertility and consumption choices [1, 2]		
**18. Demographic dividend**	Economic implication of demographic changes due to lower fertility rates [1, 9]	**14. Demographic dividend**	Economic effects of changes in demographic composition of society.
**19. Employment in society**	Increased workforce supply and productivity due to better child survival, reduced caregiver absenteeism and improved cognition/ education [1, 9]	**15. Employment in society**	Impact on overall employment in society.
**20. Consumption**	Increased consumption due to reduced morbidity and mortality [9]	**16. Impact on consumption behaviour**	Impact on the consumption of the general population.
**21. GDP**	Increased GDP due to increased consumption and productivity [9]	**17. Impact on gross domestic product (GDP)**	Impact on gross domestic product in general.
**22. Tax revenue**	Increased tax revenue due to lower health care expenditure, increased consumption and increased productivity [9]	**18. Impact on tax revenue**	Impact on tax revenue

1. Ozawa, S., et al., *Cost-effectiveness and economic benefits of vaccines in low-and middle-income countries: A systematic review.* Vaccine, 2012: p. 96-108.
2. Bärnighausen, T., et al., *Accounting for the full benefits of childhood vaccination in South Africa.* South African Medical Journal, 2008. **98**(11): p. 842-.
3. Dickson, R., et al., *Effects of treatment for intestinal helminth infection on growth and cognitive performance in children: systematic review of randomised trials.* BMJ, 2000. **320**(7251): p. 1697-1701.
4. Sakti, H., et al., *Evidence for an association between hookworm infection and cognitive function in Indonesian school children.* Tropical Medicine & International Health, 1999. **4**(5): p. 322-334.
5. Beutels, P., N. Thiry, and P. Van Damme, *Convincing or confusing?: Economic evaluations of childhood pneumococcal conjugate vaccination–a review (2002–2006).* Vaccine, 2007. **25**(8): p. 1355-1367.
6. World Health Organization. *Equity*. 2014 [cited 2014 10th January]; Available from: http://www.who.int/healthsystems/topics/equity/en/.
7. Shearley, A.E., *The societal value of vaccination in developing countries.* Vaccine, 1999. **17**: p. S109-S112.
8. Banerji, D., *Serious crisis in the practice of International health by the World Health Organization: The Commission on Social Determinants of Health.* International Journal of Health Services, 2006. **36**(4): p. 637-650.
9. Jit, M., et al., *The broader economic impact of vacination: reviewing and appraising the strength of evidence.* BMC Medicine, 2015(13): p. 209.
